# Workplace suicide prevention: A Narrative Review of Evidence-Based strategies and Occupational Medicine’s evolving role

**DOI:** 10.1192/j.eurpsy.2026.12077

**Published:** 2026-07-31

**Authors:** C. Sridi, N. Ben Gadha, J. Ben Khelifa, R. Nakhli, I. Kacem, F. Chelly, N. Belhadj, A. Fki, M. Maoua

**Affiliations:** 1 Occupational Medicine Department, Sahloul University Hospital; 2 University of Sousse, Faculty of Medicine of Sousse; 3 Research Laboratory LR19SP03; 4 Clinical Psychology Unit of Sahloul University Hospital; 5 Occupational Medicine Department, Farhat Hached University Hospital, Sousse, Tunisia

## Abstract

**Introduction:**

Suicide accounts for over 700,000 deaths annually worldwide, with workplaces increasingly recognized as critical settings for prevention. Occupational physicians (OPs) serve as frontline identifiers of mental health risks, yet comprehensive frameworks integrating their role into suicide prevention remain underdeveloped.

**Objectives:**

This review synthesizes evidence on workplace suicide prevention strategies, emphasizing OPs’ unique position within multidisciplinary approaches.

**Methods:**

A narrative literature review was conducted to synthesize evidence on workplace suicide prevention strategies, with emphasis on the role of occupational physicians. Three major databases (PubMed, PsycINFO, and the Cochrane Library) were systematically searched for publications between 2015 and 2024. Search terms included “workplace suicide prevention,” “occupational mental health,” “gatekeeper training,” “postvention protocols,” and “physician liaison.” Studies were included if they focused on employed populations, were peer-reviewed, and addressed suicide prevention, intervention, or postvention. Exclusion criteria covered non-occupational settings, non-suicidal self-harm, and non-English or non-French publications.

**Results:**

High-risk sectors for suicide include healthcare, construction, and emergency services, where job strain, trauma exposure, and stigma significantly elevate risk. Universal interventions like mental health literacy programs effectively reduce stigma by thirty to forty percent. Selective strategies, such as gatekeeper training, improve early identification of suicidal ideation by fifty percent. Indicated interventions led by occupational physicians, including screening tools like PHQ-9 and C-SSRS, increase referral rates to psychiatric services by sixty-five percent. Occupational physicians serve as critical gatekeepers, bridging employees to psychiatric care with median referral times of twenty-four hours in proactive models. They also function as system integrators, coordinating with employee assistance programs and mental health services, and lead postvention efforts to mitigate traumatic impact.

**Conclusions:**

Occupational physicians are central to effective workplace suicide prevention as gatekeepers, crisis coordinators, and system integrators. Prioritizing standardized training, integrated protocols with psychiatric services, and policy mandates in high-risk sectors will transform workplaces into key platforms for mental health crisis mitigation, making suicide prevention an urgent public health priority in occupational settings.

**Disclosure of Interest:**

None Declared

